# Ginsenoside Rg1 Induces Autophagy in Colorectal Cancer through Inhibition of the Akt/mTOR/p70S6K Pathway

**DOI:** 10.4014/jmb.2310.10043

**Published:** 2024-01-12

**Authors:** Ruiqi Liu, Bin Zhang, Shuting Zou, Li Cui, Lin Lin, Lingchang Li

**Affiliations:** 1 Jiangsu Province Academy of Traditional Chinese Medicine, 210028 Nanjing, Jiangsu Province, P.R. China; 2 Nanjing Lishui District Hospital of Traditional Chinese Medicine, 211200 Nanjing, Jiangsu Province, P.R. China; 3 Gastroenterology, Shenzhen Hospital of Guangzhou University of Chinese Medicine, 518000 Shenzhen, Guangdong Province, P.R. China

**Keywords:** Ginsenoside Rg1, autophagy, colon cancer, Akt/mTOR/p70S6K pathway

## Abstract

This study aimed to elucidate the anti-colon cancer mechanism of ginsenoside Rg1 in vitro and in vivo. Cell viability rate was detected using the 3-(4,5-dimethylthiazol-2-yl)-2,5-diphenyltetrazolium bromide (MTT) tetrazolium assay. The inhibitory effect of ginsenoside Rg1 against CT26 cell proliferation gradually increased with increasing concentration. The in vivo experiments also demonstrated an antitumor effect. The monodansylcadaverine (MDC), transmission electron microscopy (TEM), and expression of autophagy marker proteins confirmed that ginsenoside Rg1 induced autophagy in vitro. Ginsenoside Rg1 induced autophagy death of CT26 cells, but this effect could be diminished by autophagy inhibitor (3-methyladenine, 3-MA). Additionally, in a xenograft model, immunohistochemical analysis of tumor tissues showed that the LC3 and Beclin-1 proteins were highly expressed in the tumors from the ginsenoside Rg1-treated nude mice, confirming that ginsenoside Rg1 also induced autophagy in vivo. Furthermoer, both in vivo and in vitro, the protein expressions of p-Akt, p-mTOR, and p-p70S6K were inhibited by ginsenoside Rg1, which was verified by Akt inhibitors. These results indicated that the mechanism of ginsenoside Rg1 against colon cancer was associated with autophagy through inhibition of the Akt/mTOR/p70S6K signaling pathway.

## Introduction

Colorectal cancer is a common malignant tumor, causing almost 900,000 deaths annually [1]. Its morbidity rate ranks third in the world and its mortality rate ranks second in the world. Currently, oxaliplatin, 5-Fu, and irinotecan are the main drugs used to treat colon cancer. However, serious gastrointestinal side effects and neurotoxicity associated with these drugs significantly affect the quality of life in patients with colon cancer. Therefore, research on high-efficiency and low-toxicity drugs for colon cancer has become a hot topic. Studies have showed that *Panax ginseng* has many beneficial properties, including anti-cancer, anti-diabetic, and anti-inflammatory effects [2]. One of the main active components in this plant is ginsenoside, which can be divided into ginsenoside Rb1, Rg3, Rd, Rg1, Rh2, Rc, Rb2, Re, Rf, F1, PPD, PPT, and compound K [3]. Compared to other ginsenosides, ginsenoside Rg1 has the advantages of multi-target effects and few side effects [4]. Ginsenoside Rg1 has also been shown to exhibit anti-cancer activity. It exerts clear inhibitory effects on many types of cancers, such as breast cancer [5], nasopharyngeal cancer [6], and osteosarcoma [7]. We investigated whether ginsenoside Rg1 has anti-colon cancer potential.

Autophagy is closely related to the occurrence of tumors, and can highly regulate the catabolic process of cells [8]. Autophagy eliminates damaged proteins, organelles, and pathogens. The Akt/mTOR pathway can be activated during the development of a variety of human cancers and is considered a relevant therapeutic target [9]. Autophagy can be regulated through this pathway. PI3K, a kinase that represents that most upstream molecule of autophagy, can trigger autophagy signals and activate mTORC1 [10]. The mTOR kinase is an important regulatory molecule of autophagy, and activated mTOR can inhibit autophagy. In addition, the p70S6K target can be activated by the mTOR pathway to regulate autophagy. Therefore, the Akt/mTOR/p70S6K pathway is closely related to autophagy and may be a potential target for cancer treatment.

In this study, the anti-colon cancer potential of ginsenoside Rg1 was the first to investigate. To further study whether the anti-colon cancer mechanism is related to autophagy induction, and combined with the classic autophagy pathway to research the mechanism of autophagy induction. This article provides references for future research on the anti-cancer mechanism of ginsenoside Rg1. It provides theoretical basis for clinical search of high efficiency and low toxicity anti-colon cancer drugs.

## Materials and Methods

### Reagent and Chemicals

Ginsenoside Rg1 (≥ 98.0%, HPLC) was purchased from Shanghai yuanye Bio-Technology Co., Ltd. (China). Rapamycin (092220201231) was purchased from Shanghai Beyotime Biotechnology Co., Ltd. The monodansylcadaverine (20210312) was obtained from Keygen Biotech Co., Ltd. (China). Antibodies against Akt (BS-6951R), p-Akt (BSM-33281M), mTOR (BS-1992R), p-mTOR (BM4840), p70S6K (BS-1426R), p-p70S6K (9234T), Beclin-1 (BSM-33323M), LC3-II (BS-8878R), and β-actin (SC-8432) were purchased from Santa Cruz Biotechnology (USA). Fetal bovine serum (FBS) (21020702) and RPMI 1640 (20210114) incomplete medium were purchased from Sigma (USA).

### Equipment

Electronic balance (YH-C1002, Ruian Yingheng Electric Appliance Co., Ltd.,); Rotary evaporation instrument (RE-5203, Shanghai Yarong Biochemical Instrument Factory); Vacuum sterilization pot (GI80DP, Guangzhou Shenhua Biotechnology Co., Ltd.); Digital display constant temperature stirring circulating water tank (HH-60, Changzhou Guohua Electric Appliance Co., Ltd.); Biosafety cabinet (1300 Series II, Thermo Fisher (Suzhou) Instrument Co., Ltd); Table centrifuge (TDL-40B, Shanghai Anting Scientific Instrument Factory); Inverted microscope (XDS-1B, Chongqing Photoelectric Instrument Co., Ltd.); CO_2_ cell incubator (HERAcell150, Thermo Forma); -80°C Freezer (-86C ULT Freezer, Thermo Forma); Fluorescent inverted microscope (Olympus IX73, Nanjing Aoli Scientific Instrument Co., Ltd.); Transmission electron microscopy (JEM-1400, Electronics Co., Ltd., Japan).

### Cell Culture

Murine colon cancer CT26 cells were preserved in the Molecular Biology Laboratory of Jiangsu Institutes of TCM. The CT26 cells were cultured in RPMI-1640 incomplete medium containing 10% heat-inactivated FBS, 80 U/ml penicillin, and 0.08 mg/ml streptomycin. Cells were kept at 37°C in a 5% CO_2_ constant-temperature cell incubator.

### Animals

Male nude mice (BALB/c nude, 5-week-old) were purchased from Bengbu Yinuojia Biotechnology Co., Ltd., and raised under specific pathogen free (SPF) conditions in the animal management institution with a photoperiod of 12:12 h, ambient temperature of 22–24°C, and relative humidity of 45%. The mice were allowed to plenty of water and food. Animal procedures were conducted under approved guidelines by the Ethics Committee of Jiangsu Province Hospital on Integration of Chinese and Western Medicine (NO. AEWC-20230918-330), and follow the National Research Council's Guide.

### Cell Viability Assay

The cell concentration was diluted to 5 × 10^4^ cells/ml, and 100 μl of diluted cell suspension was added to each well of a sterile 96-well plate. When the cells grew to 80% density, they were treated with different concentrations of ginsenoside Rg1 for 24 h. Then, 50 μl of MTT solution was added to each well. After 4 h of incubation, 150 μl dimethylsulfoxide(DMSO) was added to each well and wobbled on a plate shaker for 10 min to completely dissolve the crystals. The absorbance was detected at 490 nm using a microplate reader.

### Autophagy Staining Assay Kit with MDC

The cell concentration was diluted to 1 × 10^5^ cells/ml, and the climbing pieces were placed in a 24-well plate; then, 500 μl of RPMI-1640 incomplete medium was added to wet the bottom of the dish, and 500 μl of the cell suspension was slowly added along the well wall. When the cells grew to 80% density, the cells were treated with different concentrations of ginsenoside Rg1 and rapamycin for 24 h. The cells were then rinsed twice in 1× wash buffer, 100 μl of MDC working solution was added, and the cells were stained at 25°C for 30 min in darkness. After aspirating the original culture, the cells were rinsed thrice in 1× wash buffer. The fluorescence microscope was used to observe the dyed cells.

### Transmission Electron Microscopy

The cell concentration was diluted to 2 × 10^5^ cells/ml; then, 500 μl of the diluted cell suspension transferred to individual wells of a sterile 6-well plate. When the cells grew to 80% density, ginsenoside Rg1 of different concentrations was added to the wells. Cell precipitation was obtained after digestion and washing. The cells were fixed with 2.5% glutaraldehyde and phosphate buffer for at least 2 h and then dehydrated in ethanol and acetone. A mixture of pure acetone and embedding solution (2:1) was added to the cells at room temperature for 4 h, and allowed to treat the cells overnight in a 37°C oven. Lkb-1 ultra-thin slicer was used to cut the tissue into slices of 50–60 nm, uranyl acetate and lead citrate were used for staining, and the images were acquired using a transmission electron microscope.

### Western Blot Assay

The cell concentration was diluted to 1 × 10^5^ cells/ml. Then, 1 ml of the cell suspension was transferred into a sterile 6-well plate. When cells reached 80% density, different concentrations of ginsenoside Rg1 were added to the wells for 24 h. Cell precipitation was obtained after digestion and washing. To extract the protein, a lysate of 5 times the volume of the precipitate was added, and the precipitate was removed. It was extracted at 4°C for 1 h using ultrasonic treatment; centrifugation was carried out at 10000 r/min for 10 min, and the supernatant was collected. The tumor tissue was cut into small pieces with scissors and completely ground into powder, then transferred to pre-cooled EP tubes and thoroughly mixed with protein lysate. Cells or tumor tissues were lysed in RIPA buffer to obtain protein samples. Membranes were incubated with the corresponding primary antibodies overnight at 4°C and then with the secondary antibody for 2 h. The enhanced chemiluminescence (ECL) was used for band detection.

### In Vivo Xenograft Analysis

To establish a mouse xenograft tumor model, the CT26 cell suspension (2 × 10^5^ cells/100 μl) was inoculated subcutaneously on the flank of the nude mice. Changes in the tumor volume was observed daily using the formula (1/2[L×W^2^]). After the tumor volume reached 50~100 mm^3^, the mice were randomly divided into three groups, six in each group: negative control, positive control, and ginsenoside Rg1 group. In the positive control group, 5-FU (20 mg/kg) was injected intraperitoneally every 3 d. The mice in the ginsenoside Rg1 group were intraperitoneally injected with ginsenoside Rg1 (100 mg/kg) once a day, and normal saline was injected intraperitoneally every day in the negative control group. The dosing period was 14 days. At the end of the experiment, all mice were sacrificed by cervical dislocation. Tumor tissues were isolated, weighed and fixed using 4% paraformaldehyde.

### Immunohistochemistry Assay

The tumor tissues were carefully fixed in 4% paraformaldehyde (20 times the volume of the tumor) for 2 d. Then, the tumor tissue was embedded in paraffin and the sections were prepared. Sections were routinely dewaxed in water and antigen-repaired 5% Bovine serum albumin(BSA) blocking solution was added and the sections were incubated at 37°C for 30 min. The sections were incubated with Beclin-1 and LC3-II antibodies at 4°C overnight and then incubated with the secondary antibodies. DAB(diaminobenzidine tetrahydrochloride) developer solution was used for color rendering, and Mayer’s hematoxylin was added for redyeing. Neutral gum-sealing was performed, three fields in each group were randomly selected under a 200 × light microscope for image acquisition. The presence of brownish yellow particles on the tissue is a positive area. ImageProplus was used to calculate the average optical density of the positive region of immunohistochemical tissues.

### Statistical Analysis

Data were obtained from at least three independent experiments and expressed as the mean ± standard deviation (SD). Student’s t-test or one-way analysis of variance (ANOVA) was used for statistical comparisons. Statistical significance was set at *p* < 0.05.

## Results

### Ginsenoside Rg1 Inhibited Cell Proliferation of CT26 Cells

**Fig. 1:**
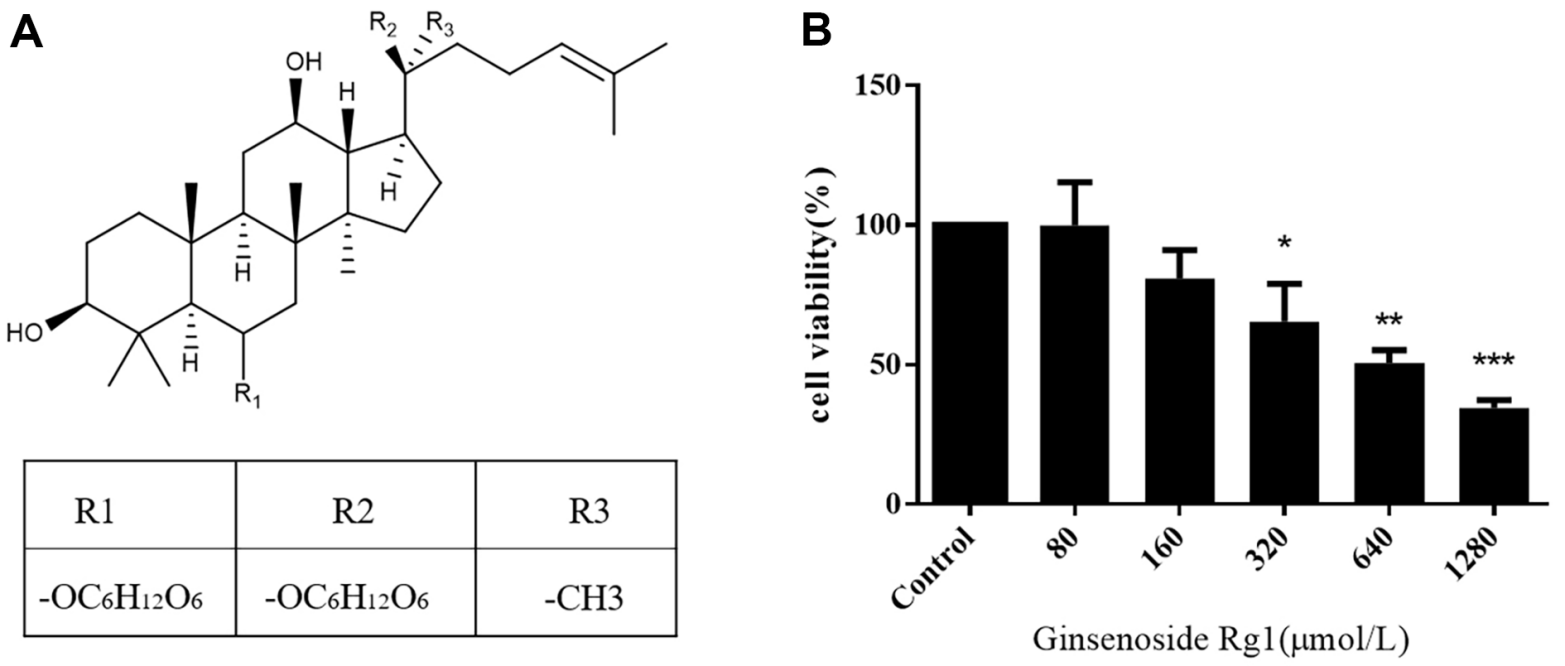
Ginsenoside Rg1 inhibits the growth of colon cancer cells. (**A**) The structure of Ginsenoside Rg1. (**B**) CT26 cells were incubated with different concentrations of ginsenoside Rg1 for 24 h. Ginsenoside Rg1 inhibited the proliferation of CT26 cells in a dose-dependent manner. *P < 0.05, **P < 0.01, vs. control group.

CT26 cells were exposed to different concentrations of ginsenoside Rg1 to explore its survival rates using MTT assay. Compared with the control group, the cell proliferation was decreased by 50% after treatment with 640 μmol/L ginsenoside Rg1. Ginsenoside Rg1 inhibited the proliferation of CT26 cells in a dose-dependent manner (Fig. 1). Thus, ginsenoside Rg1 has an anti-cancer effect in colon cancer cells.

### Ginsenoside Rg1 Induced Autophagy in CT26 Cells

**Fig. 2:**
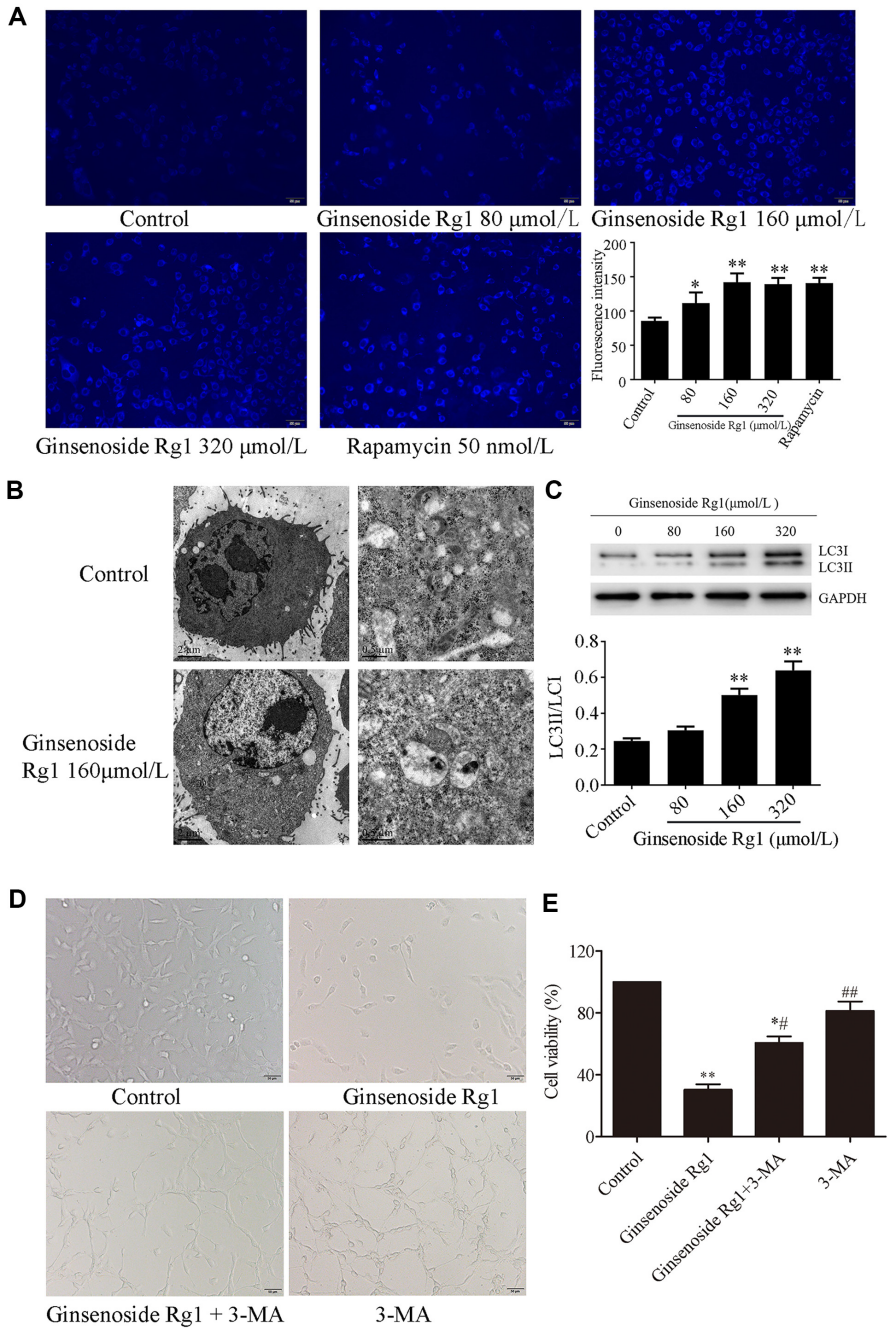
Ginsenoside Rg1 induces autophagy in colon cancer cells. (**A**) MDC method (×200). The autophagosomes emitted a blue-green fluorescence under the fluorescence microscope. (**B**) TEM images. Scale bar, 2 um and 0.5 um. Autophagy lysosome-like structures were observed in the cells of the ginsenoside Rg1 group. (**C**) The expression of LC3 protein in CT26 cells. LC3 is the autophagy marker proteins, was highly expressed in the ginsenoside Rg1 treated group. (**D**) CT26 cell morphology was observed under inverted fluorescence microscope after treated with 3-MA. (**E**) MTT was used to measure the cell survival rate after treatment with 3-MA. 3-MA was used to block cell autophagy. The inhibition of autophagy markedly restored cell growth in ginsenoside Rg1-treated cells. *P < 0.05, **P < 0.01, vs. control group; ^#^*P* < 0.05, ^##^*P* < 0.01, vs. ginsenoside Rg1 group.

Based on the results of the MTT assay, the concentrations of ginsenoside Rg1, namely 80, 160, and 320 μmol/l, were chosen for the following experiment. In order to confirm whether ginsenoside Rg1 induced autophagy in CT26 cells, the formation of autolysosomes was observed after staining with MDC. As shown in Fig. 2A, the autophagosomes emitted a blue-green fluorescence under the fluorescence microscope. Compared with the control group, the fluorescence intensity of the cells in the ginsenoside Rg1 group was significantly enhanced (*p* < 0.05, *p* < 0.01). Furthermore, the structure of autophagosomes was observed using TEM. As shown in Fig. 2B, autophagy lysosome-like structures were observed in the cells of the ginsenoside Rg1 group. The degraded organelles in autophagosomes were observed after magnification. To further verify whether ginsenoside Rg1 induced autophagy, western blotting assay was used to detect the expression of autophagy-associated proteins. As seen in Fig. 2C, compared with the control group, the ratio of the protein expression of LC3II/LC3I was significantly increased in the ginsenoside Rg1 160 and 320 μmol/l groups (*p* < 0.01). Then, the 3-methyladenine (3-MA, an autophagy inhibitor) was used to investigate whether autophagy was relevant in the anticancer effect of ginsenoside Rg1. The morphological changes were observed using inverted microscope. As shown in Fig. 2D, cells in the control group had a well extended shape and clear boundaries. However, after treatment with ginsenoside Rg1, the number of adherent cells decreased and a few cells changed their shape from slender to round. After treatment with ginsenoside Rg1 and 3-MA, the number of adherent cells was increased and the morphological characteristic was changed from atrophy to extended state. The cells treated with 3-MA were well extended and only a few cells were shrunken. The cell viability was also determined by MTT after ginsenoside Rg1 or 3-MA treatment. The cell viability in the 3-MA group and control group was almost the same. However, compared with the control group, the cell viability was significantly decreased in the ginsenoside Rg1 group (*p* < 0.01). The reduced cell viability was reversed by ginsenoside Rg1 + 3-MA (Fig. 2E). These data suggest that the inhibition of ginsenoside Rg1 on colon cancer cell growth was associated with autophagy.

### Ginsenoside Rg1 Inhibited Akt/mTOR/p70S6K Signaling Pathway In Vitro

**Fig. 3:**
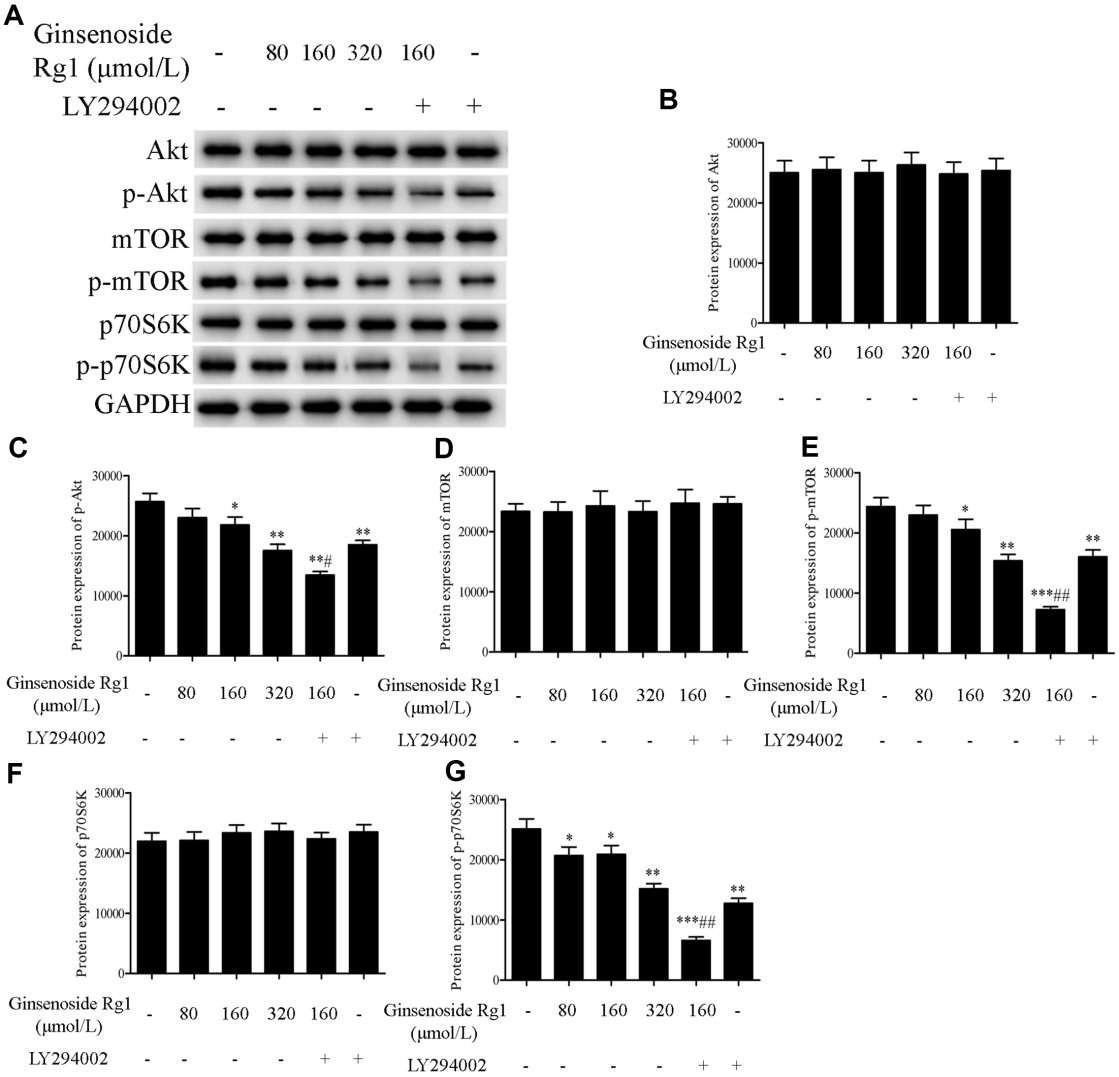
Effect of ginsenoside Rg1 on the expressions of Akt/mTOR/p70S6K pathway-related proteins in CT26 cells. The Akt/mTOR/p70S6K pathway is closely related to the occurrence of autophagy. Moreover, LY294002 (an Akt inhibitor) was adopted to verify the inhibitory effect of ginsenoside Rg1 on Akt/mTOR/p70S6K signaling pathway. *P < 0.05, **P < 0.01, ***P < 0.001, vs. control group; ^#^*P* < 0.05, ^##^*P* < 0.01, vs. ginsenoside Rg1 160 μmol/l group.

The protein expressions of Akt, p-Akt, mTOR, p-mTOR, p70S6K, p-p70S6K in CT26 cells were detected using the western blotting assay. As illustrated in Fig. 3, compared with the control group, ginsenoside Rg1 reduced the protein expressions of p-Akt, p-mTOR, and p-p70S6K (*p* < 0.05, *p* < 0.01), but the protein expressions of Akt, mTOR, and p70S6K remained unaltered. Moreover, LY294002 (an Akt inhibitor) was adopted to verify the inhibitory effect of ginsenoside Rg1 on Akt/mTOR/p70S6K signaling pathway. The inhibitory action of the combination of ginsenoside Rg1 and LY294002 on the protein expressions of p-Akt, p-mTOR, and p-p70S6K was stronger than that in the ginsenoside Rg1 group (*p* < 0.05, *p* < 0.01). Thus, ginsenoside Rg1 may induce autophagy in CT26 cells by inhibiting the Akt/mTOR/p70S6K signaling pathway.

### Ginsenoside Rg1 Inhibited the Growth of Xenograft Tumor in BALB/c Mice

**Fig. 4:**
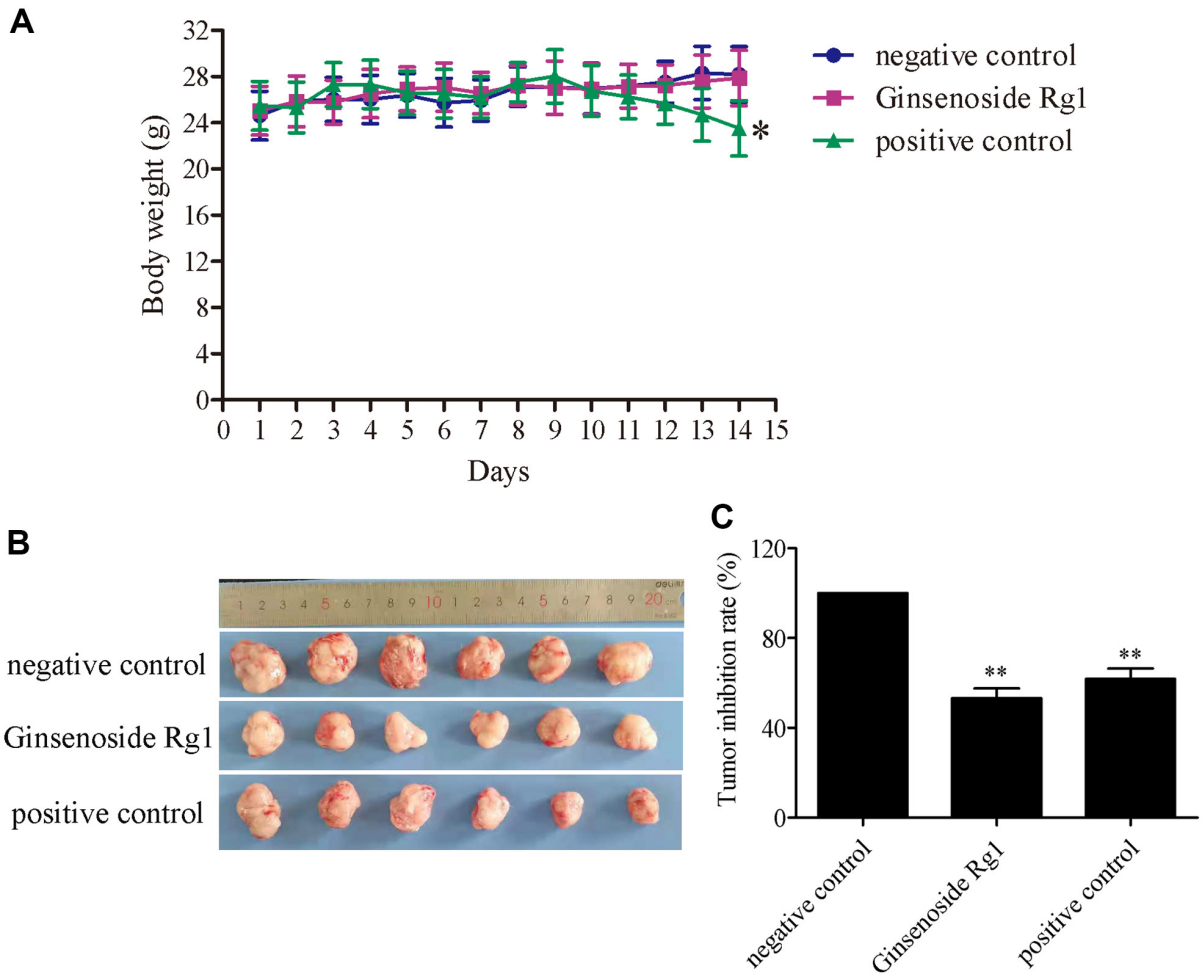
The Antitumor effect of ginsenoside Rg1 in vivo. (**A**) The body weight of nude mice during the treatment days. (**B**) Images of tumor tissues. (**C**) Tumor inhibition rate. *P < 0.05, **P < 0.01, vs. negative control group.

Ginsenoside Rg1 inhibited the proliferation of CT26 cells in vitro. We then determined whether ginsenoside Rg1 inhibited the growth of colon cancer in vivo. As shown in Fig. 4A, the weight of nude mice in the negative control and ginsenoside Rg1 groups slightly increased within 14 days. However, the weight of the nude mice in the positive group decreased from the day 9 to day14. After 14 days, the tumor tissue was obtained, and tumor size in the ginsenoside Rg1 group was smaller than that in the negative control group. As shown in Fig. 4C, compared with the negative control group, the tumor inhibition rate of 100 mg/kg ginsenoside Rg1 was 53.1%, with a tumor inhibition rate of 61.7% for the positive group. This indicates that ginsenoside Rg1 can inhibit tumor growth in vivo.

### Ginsenoside Rg1 Induced Autophagy and Inhibited Akt/mTOR/p70S6k Pathway In Vivo

**Fig. 5:**
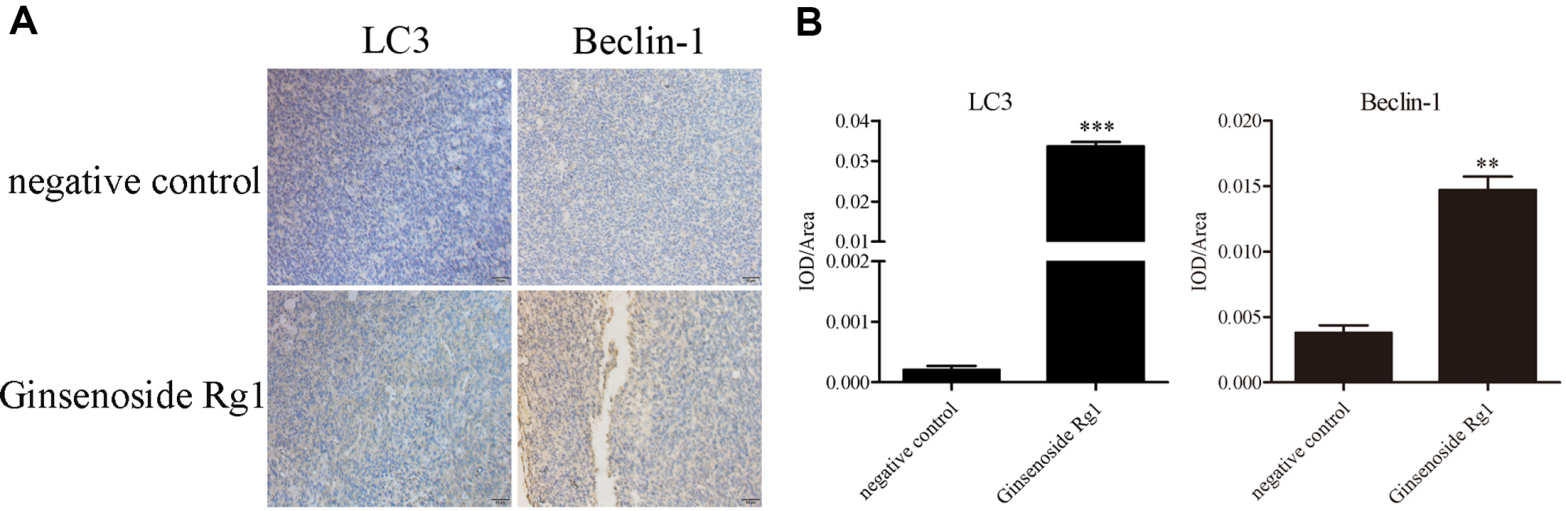
Effect of ginsenoside Rg1 on the expression of autophagy-associated proteins in tumor tissues. (**A**) Immunohistochemical analysis of LC3 and Beclin-1. LC3 and Beclin-1 are the autophagy marker proteins. Its high expression indicates the occurrence of autophagy. (**B**) Quantitative analysis. **P < 0.01,***P < 0.001, vs. negative control group.

**Fig. 6:**
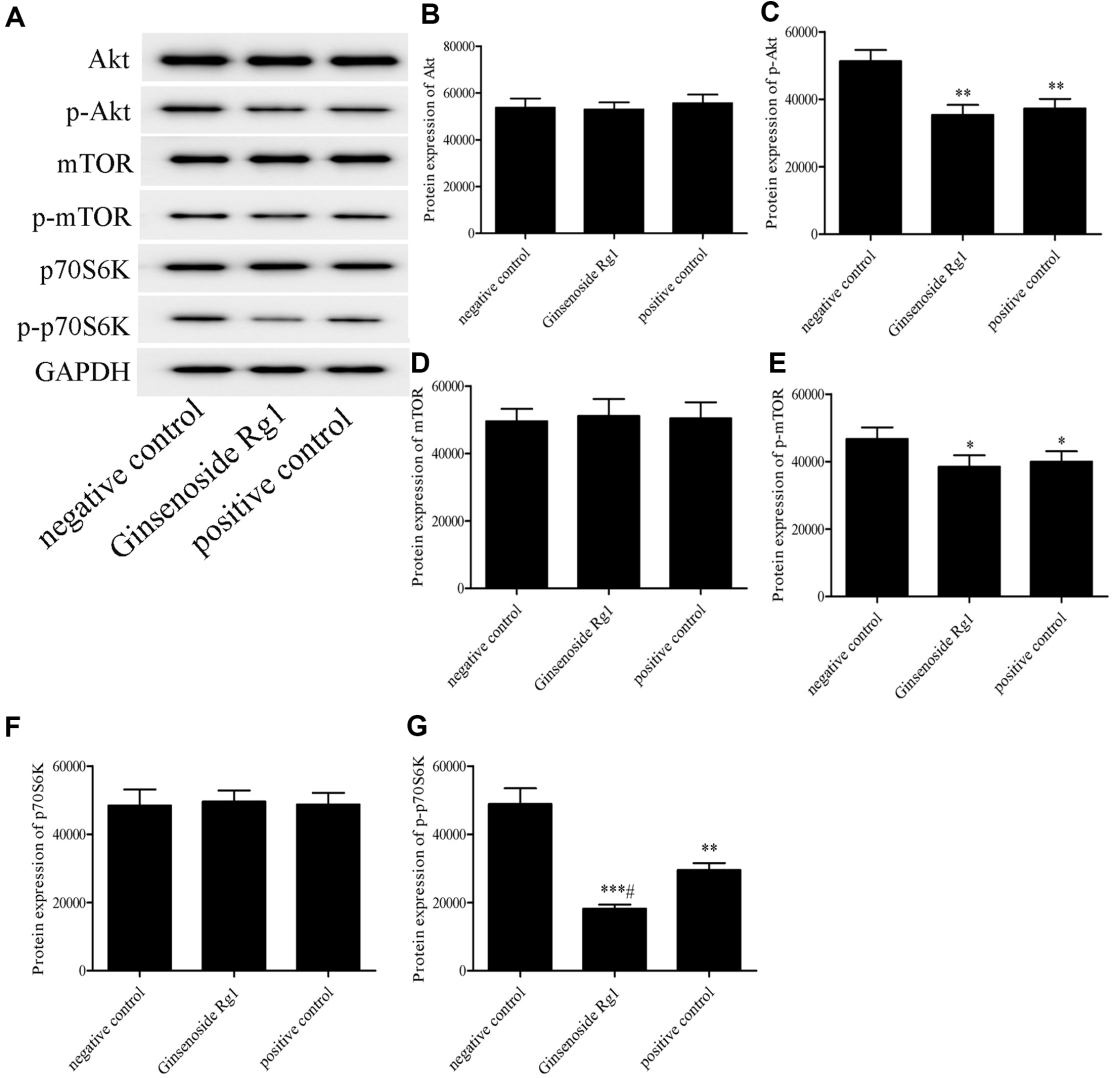
Effect of ginsenoside Rg1 on the expressions of Akt/mTOR/p70S6K pathway-related proteins in xenograft tumors. *P < 0.05, **P < 0.01, vs. negative control group; ^#^*P* < 0.05, vs. positive control group.

To detect the expression of autophagy marker proteins in tumors, immunohistochemistry assay was carried out to evaluate the protein expressions of LC3 and Beclin-1. As shown in Fig. 5, compared with the negative control group, there were abundant brownish yellow particles in the tumor sections from mice treated with ginsenoside Rg1. The autophagy marker proteins, LC3 and Beclin-1, were highly expressed in the ginsenoside Rg1 treated group. Thus, ginsenoside Rg1 induced autophagy in vivo. Then, western blotting assay was performed to further explore the effects of ginsenoside Rg1 on the expressions of Akt/mTOR/p70S6K pathways-related proteins in vivo. Compared with the negative control group, ginsenoside Rg1 groups decreased the expressions of p-Akt, p-mTOR, and p-p70S6k proteins, but ginsenoside Rg1 had little effect on the protein levels of Akt, mTOR, and p70S6k (Fig. 6). This result is consistent with in vitro studies.

## Discussion

In the recent decades, cancer has become a serious threat to people’s lives. In clinical, chemotherapy, radiotherapy, surgery, and hormone therapy are the primary methods to treat cancer [11]. However, the toxicity and side effects seriously affect the treatment and prognosis of patients. Therefore, the search for safe and effective drugs from traditional Chinese medicine attracted wide interest of researchers. *P. ginseng* is an araliaceous perennial herb with anti-inflammatory, anti-cancer and antioxidant effects. Studies have shown that ginsenoside Rg3 is an effective antitumor drug that can inhibit metastasis in colon cancer cells by inhibiting epithelial-mesenchymal transition (EMT) [12]. Ginsenoside Rh4 induces autophagy-mediated colorectal cancer cell death by activating the ROS/JNK/p53 pathway [13]. Ginsenoside Rg1 has been shown to exhibit anticancer potential, but its mechanism of action against colon cancer is not well understood. We investigated the effect of ginsenoside Rg1 against colon cancer in vivo and in vitro. The MTT assay revealed that ginsenoside Rg1 had a significant inhibitory effect on the viability of CT26 cells. In a mice xenograft tumor model, ginsenoside Rg1 inhibited the growth of xenograft colon cancer tumors. Our results indicated that ginsenoside Rg1 had a significant inhibitory effect of colon cancer both in vitro and in vivo.

Autophagy is a versatile degradation system for maintaining cellular homeostasis whereby damaged organelles and misfolded proteins are encapsulated in autophagosomes, which then combine with lysosomes to degrade these organelles [14]. Research findings have shown that autophagy was related to cell death, and its potential to regulate cell death highlighted a new strategy to treat cancer. Aloe-emodin induces autophagy and cell death by activating MAPK signaling and inhibiting Akt/mTOR pathway [14]. The combination of aloin and metformin induces apoptosis and autophagy in hepatocellular carcinoma, thereby enhancing the antitumor effect [15]. Babaodan capsules can inhibit the growth of non-small cell lung cancer (NSCLC) cells by inducing autophagy to achieve anti-tumor effects [16]. In our study, we found CT26 cells manifested an autophagic lysosome structure, the fluorescence intensity was enhanced, and the expression of LC3II/LC3I was upregulated in CT26 cells after treatment with ginsenoside Rg1. Immunohistochemical results showed that the expression of LC3 and Beclin-1 proteins in the tumors of nude mice from the ginsenoside Rg1 group was higher than those in the negative control group. These results suggest that the anticancer activity of ginsenoside Rg1 may be related to the activation of autophagy both in vitro and in vivo. However, autophagy, as a major cellular degradation mechanism, can inhibit or promote tumor growth, or even play a neutral role [8]. Autophagy could block the proliferation of cancer cells by eliminating tumorigenic protein substrates and damaged organelles. On the other hand, cancer cells meet the metabolic needs of proliferating tumor cells through autophagy to promote tumor growth [17]. To explore the relationship between autophagy and ginsenoside Rg1-induced cancer cell death, CT26 cells were treated with 3-MA, which was used to block cell autophagy. The inhibition of autophagy markedly restored cell growth in ginsenoside Rg1-treated cells. These results indicated that ginsenoside Rg1 induced autophagy in colon cancer cells to block the proliferation of cancer cells.

Autophagy is regulated by related kinase targets and kinase-mediated phosphorylation, such as Akt, AMPK, and mTOR. The phosphorylated Akt inhibits TSC1/2, leading to mTORC1 activation. mTORC1 inhibits the formation of autophagosomes by inactivating the formed autophagic regulatory complex through phosphorylation. These autophagy-related kinases interact to form signaling pathways [10]. Autophagy related pathways regulate cell proliferation, growth, metabolism and metastasis. The Akt/mTOR pathway has been widely studied in tumorigenesis and is generally activated in cancer [18]. Studies have shown that E2F2 regulates autophagy through the PI3K/Akt/mTOR pathway, which in turn affects the migration of gastric cancer cells [19]. Echinatin inhibits the growth and invasion of esophageal squamous cell carcinoma by inactivating the Akt/mTOR signaling pathway [20]. Sinomenine regulates autophagy via the PI3K/Akt/mTOR pathway and plays an anti-melanoma role [21]. In addition, the p70S6K target, which lies downstream of mTOR, can be activated by the mTOR pathway to regulate autophagy. Therefore, the Akt/mTOR/p70S6K pathway is closely related to the occurrence of autophagy, and the regulation of autophagy via inhibiting this pathway represents a strategy for the treatment of various diseases [9]. However, it has not been reported whether ginsenoside Rg1 induces autophagy via the Akt/mTOR/p70S6K pathway. In our study, compared with that the control group, the total levels of Akt, mTOR, and p70S6K proteins in CT26 cells showed little change after ginsenoside Rg1 treatment. However, the levels of phosphorylated proteins of Akt, mTOR, and p70S6K were significantly decreased in ginsenoside Rg1 group. LY294002 (an Akt inhibitor) demonstrated that ginsenoside Rg1 induced autophagy by inhibiting the Akt/mTOR/p70S6K signaling pathway. The results of our study are consistent with previous reports. Rubioncolin C induced apoptosis of cancer cells and autophagy-related death, while inhibitng the Akt/mTOR/p70S6K signaling pathway [22]. Caffeine induced autophagy by interfering with the Akt/mTOR/p70S6K pathway [23]. Delicaflavone could induce autophagic cell death by interfering with the Akt/mTOR/p70S6K signaling pathway [24]. We further studied the effects of ginsenoside Rg1 on Akt/mTOR/p70S6K pathway *in vivo*. Western blotting assay showed that ginsenoside Rg1 could remarkably decrease the expressions of p-Akt, p-mTOR, p-p70S6k protein in a xenograft model. Thus, ginsenoside Rg1 induced autophagy via inhibiting the Akt/mTOR/p70S6K pathway in colon cancer.

## Conclusion

In this study, the anti-colon cancer effect of ginsenoside Rg1 and its mechanism of action were investigated. Ginsenoside Rg1 inhibited the proliferation of CT26 cells in vitro and inhibited colon tumor growth in vivo. The possible mechanism of action was associated with the induction of autophagy via inhibiting the Akt/mTOR/p70S6K pathway. Ginsenoside Rg1 was a candidate for the treatment of colon cancer. This study provided experimental basis for clinical selection of safe and effective anti-colon cancer drugs.
